# RACIAL/ETHNIC DISPARITIES IN THE EFFECTS OF RECEIVING A DIAGNOSIS OF DEMENTIA ON SOCIAL RELATIONSHIPS

**DOI:** 10.1093/geroni/igac059.1688

**Published:** 2022-12-20

**Authors:** Takashi Amano, Addam Reynolds, Clara Scher, Yuane Jia

**Affiliations:** Rutgers University-Newark, Newark, New Jersey, United States; Rutgers University, New Brunswick, New Jersey, United States; Rutgers, The State University of New Jersey, New Brunswick, New Jersey, United States; Rutgers University, New Brunswick, New Jersey, United States

## Abstract

Although early diagnosis has been recognized as a key strategy to improve outcomes for those with dementia, receiving a diagnosis may negatively influence social relationships. Absent from the literature are considerations of how race/ethnicity may alter these associations. This study examines racial/ethnic variation in the effects of receiving a diagnosis of dementia on social relationships of older adults. Data from the three waves of the Health and Retirement Study were utilized as part of this study. This study examined whether receiving a new diagnosis of dementia changed subsequent social relationships (social networks, social engagement, social support). Regression analyses with inverse probability weighting were performed to estimate the impact of receiving a dementia diagnosis on changes in social relationships. Receiving a new diagnosis of dementia reduced both informal (b = -0.476, p = 0.011, 95% CI = [-0.842, -0.110]) and formal (IRR = 0.688, p = 0.034, 95% CI = [0.487, 0.971]) social engagements. The negative impact of receiving a diagnosis on formal social engagement was stronger among non-Hispanic Blacks (IRR = 0.047, p = 0.007, 95% CI = [0.005, 0.432]). We found no statistically significant impacts of receiving a diagnosis of dementia on social networks and social support. Results suggest that receiving a new diagnosis of dementia may have unintended negative consequences on social engagement and may be more salient for racial/ethnic minorities, such as non-Hispanic Blacks. Practitioners and policymakers should be aware of these consequences and should identify strategies to alleviate the negative implications of receiving a diagnosis.

